# Comparative Study on Combined Addition of Gd-Ce and Gd-Y on the Mechanical Properties and Electrochemical Behavior of Mg-Zn-Mn-Ca Alloys

**DOI:** 10.3390/ma18010196

**Published:** 2025-01-05

**Authors:** Ke Hu, Junru Zhou, Yan Zhou, Guoxian He, Wenhao Zhao, Jingjing Guo, Xiao Liu, Lingling Li, Fujian Guo

**Affiliations:** 1 School of Materials Science and Engineering, Guangdong Ocean University (Yangjiang Campus), Yangjiang 529500, China; hukee12345@126.com (K.H.); 18352852204@163.com (J.Z.); zyszds@outlook.com (Y.Z.); a2993130779@163.com (G.H.); 15338056507@163.com (W.Z.); 19704466745@163.com (J.G.); 2 School of Chemical Engineering, China University of Mining and Technology, Xuzhou 22100, China; 3 Changxing Yunfeng Luliao Co., Ltd., Huzhou 313000, China; 4 College of Marine Equipment and Mechanical Engineering, Jimei University, Xiamen 361021, China; 202361000266@jmu.edu.cn; 5 New Engineering Industry College, Putian University, Putian 351100, China; li-ll@ptu.edu.cn; 6 Yang Jiang Advanced Alloys Laboratory, Yangjiang 529500, China

**Keywords:** Mg-3Zn alloy, extrusion, electrochemical behavior, mechanical properties, microstructure

## Abstract

This study presents a comparative analysis of the influence of Ce-Gd and Gd-Y additions on the microstructural evolution, mechanical properties, and electrochemical behavior of extruded Mg-3Zn-Mn-Ca alloy rods. Despite the frequent incorporation of Gd, Y, and Ce as alloying elements in magnesium alloys, the systematic examination of their combined effects on Mg-Zn alloys has been limited. Our findings reveal that both Gd-Ce and Gd-Y additions significantly enhance the mechanical properties of Mg-3Zn-Mn-Ca alloy, although through differing mechanisms. Specifically, the Mg-3Zn-1Mn-0.5Ca-1Gd-0.5Ce(ZMXE3101(GdCe)) alloy exhibited a yield strength of 304.5 MPa and an elongation of 15%, achieved through dynamic recrystallization and enhanced basal texture. The grain refinement and texture strengthening resulting from the coarse second-phase particles formed by Ce-Gd played a significant role in increasing the yield strength. In contrast, the Mg-3Zn-1Mn-0.5Ca-1Gd-0.5Y (ZMXE3101(GdY)) alloy demonstrated a yield strength of 305 MPa and an elongation of 20%. The finer grains and elongated unrecrystallized grains formed by Gd-Y contributed to the elevation in yield strength. While the ductility of this alloy was slightly lower than that of Mg-3Zn-Mn-Ca without rare earth additions, it still exhibited commendable overall mechanical properties. The electrochemical test results indicate that the addition of both Gd-Ce and Gd-Y enhances the corrosion current density of Mg-3Zn-Mn-Ca alloy, attributable to the generation of numerous rare earth phase particles that function as cathodes. Compared to the ZMXE3101(GdY) alloy, ZMXE3101(GdCe) exhibits a higher equilibrium potential and significantly lower corrosion current density. This is due to the formation of a protective film during the corrosion process by Gd-Ce.

## 1. Introduction

Magnesium alloys, as one of the lightest structural metals, exhibit remarkable application potential and commercial value in aerospace, automobile manufacturing, and electronic equipment and are widely used owing to their low density, high specific strength, excellent thermal conductivity, effective electromagnetic shielding, good machinability, and outstanding vibration and shock absorption characteristics [1]. However, magnesium alloy with a hexagonal close-packed (HCP) crystal structure poses considerable challenges due to its fundamentally low symmetry, resulting in a limited number of slip systems under ambient temperature, typically basal and prismatic slips. This structural characteristic hinders uniform and continuous plastic deformation when magnesium alloys are subjected to external forces, leading to poor formability and inadequate strength and toughness, thus severely limiting their application scope. Therefore, enhancing the plastic deformation capacity of magnesium alloys, achieving effective synergies between strength and ductility, and improving their corrosion resistance are current focal points of magnesium alloy research, which hold significant importance for promoting the commercial prospects of magnesium alloys [2,3].

The mechanical properties and corrosion resistance can be significantly improved by introducing a certain quantity of alloying elements. Due to the variation in the solid solubility of Zn with temperature in Mg, the addition of Zn to Mg results in a favorable aging strengthening effect. However, when the Zn content exceeds 4 wt.%, the ductility and corrosion resistance of Mg-Zn binary alloys undergo significant reductions [4]. Manganese and calcium are two elements that can generate dispersed second-phase particles during the deformation process of magnesium alloys, pinning grain boundaries and thereby significantly refining the grain size of magnesium alloys. Both elements can enhance the corrosion resistance of magnesium alloys. Mn can remove iron impurities from magnesium alloys [5], while Ca can form a passive layer during the corrosion process of magnesium alloys [6,7], effectively mitigating corrosion of the magnesium alloy. Rare earth (RE) elements can substantially improve the microstructure of Mg alloys, weaken deformation textures [8,9], and enhance corrosion resistance [10]. In recent years, extensive research has been undertaken by scholars on the effects of RE element modification in magnesium alloys. RE elements that can significantly improve Mg alloys include Ce, La, Y, Gd, Nd, and Sm [11,12,13,14,15,16]. Among these elements, Gd has a notable effect on enhancing the strength of Mg. Furthermore, numerous studies have indicated that the addition of Gd can effectively enhance the corrosion resistance of Mg alloys. For instance, Zhiqiang Zhang et al. [17] examined the impact of Zn/Gd atomic ratios on the mechanical properties and corrosion resistance of Mg-Gd-Zn-Zr alloys and noted that optimal mechanical performance and corrosion resistance are attained with a Zn to Gd atomic ratio of 3:1. Yue Deng et al. [18] developed a new Mg-Zn-Gd-Mn-Sr alloy that exhibits moderate strength and outstanding corrosion resistance. In the field of mechanical behavior, Gd and Y are often added together to improve the mechanical properties of Mg alloys [19,20,21], while Ce is frequently used to improve the ductility and processing characteristics of Mg alloys [12,22,23]. However, there is limited research on the impact of simultaneously adding Ce-Gd and Gd-Y on the mechanical properties and corrosion resistance of Mg alloys. Therefore, based on previous studies, this paper added Ca and Mn to Mg-3Zn to refine the microstructure and comparatively study the effect of Ce-Gd and Gd-Y on the mechanical characteristics and electrochemical behavior of Mg-3Zn-Ca-Mn alloys.

## 2. Experimental Method

### 2.1. Material Preparation

In this comprehensive study, three distinctive magnesium alloys—Mg-3Zn-1Mn-0.5Ca, Mg-3Zn-1Mn-0.5Ca-1Gd-0.5Ce, and Mg-3Zn-1Mn-0.5Ca-1Gd-0.5Y—were meticulously explored. These cast billets were fabricated by the conventional mold casting method with 99.99% pure magnesium and zinc, as well as Mg-30Gd, Mg-25Y, Mg-30Ce, Mg-10Mn, and Mg-30Ca master alloys. At a tightly controlled temperature of 750 °C, the materials were melted under an inert protective atmosphere consisting of argon and 0.5% (by volume) sulfur hexafluoride (SF6) and then poured into preheated steel molds at 100 ± 10 °C for cooling. As a result, ingots were produced with a diameter of 100 mm and a height of 120 mm, while the detailed composition is presented in Table 1. To eliminate microstructural defects and enhance the internal structure of the alloys, the cast ingots were subjected to a homogenous treatment at 350 °C for 3 h, and then subsequently underwent rapid quenching in water to achieve room temperature. Subsequently, the ingots underwent further processing and treatment at 300 °C for 2 h and were finally hot-extruded at 300 °C at an extrusion ratio of 15:1 and an extrusion speed of 0.1 mm/s.

### 2.2. Microstructural Characterization 

In accordance with the ASTM B557M-10 standard [24], tensile test specimens with a diameter of 5 mm and a gauge length of 25 mm were cut from the optimized rods. The experimental samples were meticulously polished using a sequence of abrasive papers, with grit sizes varying from 100# to 400#, 800#, 1200#, 2000#, and 3000#, to achieve a smooth and refined surface. This was followed by fine mechanical polishing and chemical etching. The preparation of samples for Scanning Electron Microscope (SEM) analysis adhered strictly to the guidelines outlined in ASTM E3-11. Corrosion treatment was performed with an ethanol solution containing 4% acetic acid, aiming to reveal the microstructural characteristics of the alloy. X-ray diffraction (XRD) analysis was performed using an XRD-6100 instrument to clarify the crystalline structure and phase composition of the alloy. Additionally, an advanced Scanning Electron Microscope (SEM, Apreo 2 with EDS) was employed to examine the fracture surfaces and microstructure features after tensile testing in detail. The average grain size was determined by the linear intercept method, a crucial parameter for a comprehensive understanding of the mechanical properties of the material. The deformation texture of the alloy was determined using Electron Backscatter Diffraction (EBSD, MA-IA3 model 2016) technology, which is particularly important for comprehending the deformation and fracture mechanisms of the material.

### 2.3. Mechanical Properties Test

The mechanical properties were evaluated with an AGS-X series electronic universal testing machine at room temperature, following ASTM standards. The test rate was selected at 1 mm/min, ensuring that the tensile direction was aligned with the extrusion direction of the alloy. To ensure the reproducibility of the results, at least three tests were conducted.

### 2.4. Electrochemical Experiment

For precise measurement of Potentiodynamic Polarization curves and Electrochemical Impedance Spectroscopy (EIS), we utilized the advanced CHI660E electrochemical workstation (Shanghai Chenhua Co., Ltd., Shanghai, China). The experiments were conducted in a 3.5% NaCl solution, utilizing a three-electrode cell system specifically configured with a saturated Ag/AgCl electrode as the reference electrode, a platinum mesh as the counter electrode, and the test sample serving as the working electrode. Prior to the electrochemical experiments, all samples were meticulously polished with 1200-grit SiC abrasive paper to ensure a smooth test area, with a sample exposure area of 0.5 cm^2^ and an electrolyte volume of 350 mL set for the tests.

The experimental procedure was as follows: Initially, a 30 min Open-Circuit Potential (OCP) stabilization measurement was conducted to obtain the initial potential state of the samples. Subsequently, Potentiodynamic Polarization Technique (PPT) testing was performed within a voltage range of ±0.5 V relative to the free corrosion potential, using a scan rate of 0.2 mV/s. In the Potentiodynamic Polarization curves, the linear portion of the cathodic branch, starting approximately 50 mV from the corrosion potential, was selected to estimate the corrosion current density by calculating the intersection of this line segment with a vertical line drawn at the corrosion potential. After maintaining the OCP for an additional 5 min, the sample was immersed in water, and EIS measurements were conducted under open-circuit conditions. The measurement parameters were set with an AC signal amplitude of ±10 mV rms and a frequency range extending from 0.1 Hz to 30,000 Hz.

The acquired EIS spectral data were fitted and analyzed using Zview software (Version 3.1) provided by Scribner Associates Inc. (USA) to obtain the electrochemical performance parameters of the material. To ensure the reliability and accuracy of the experimental findings, all aforementioned testing steps were conducted in triplicate. The final research data were taken as three times the average of the obtained results from the three experiments. This experimental design not only ensured the precision of the data but also enhanced the reliability of the experimental outcomes.

## 3. Results and Discussion

### 3.1. Microstructure

**Figure 1:**
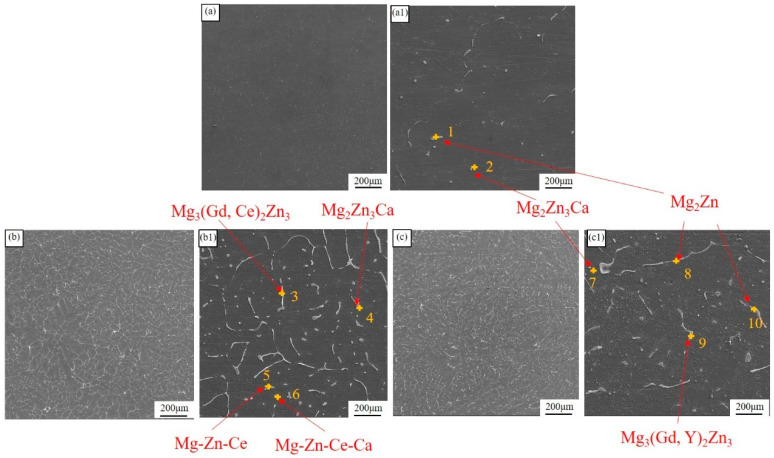
SEM micrographs of as-cast ingots. (**a**,**a1**) ZMX310; (**b**,**b1**) ZMXE3101(GdCe); (**c**,**c1**) ZMXE3101(GdY).

Figure 1 depicts the SEM morphologies of as-cast ingots. As shown in the figures, the second-phase density in ZMX310 alloy is relatively low, but it increases significantly upon the addition of RE elements. Through EDS analysis (Table 2), the ZMX310 alloy is found to primarily consist of punctuate Mg_2_Zn_3_Ca phases and rod-like Mg_2_Zn phases. After the incorporation of Gd and Ce, striped Mg_3_(Gd, Ce)_2_Zn_3_ phases, punctuate Mg-Zn-Ca-Ce phases, and rod-like Mg-Zn-Ce phases appear in the Mg matrix. In contrast, within the ZMXE3101(GdY) alloy, the Mg_3_(Gd, Ce)_2_Zn_3_ phases exhibit a disk-like morphology, while the Mg_2_Zn phases appear as stripes and coarse Mg_3_(Gd, Y)_2_Zn_3_ phases, indicating a notable precipitation of Zn from Mg upon the addition of Gd and Y.

**Figure 2:**
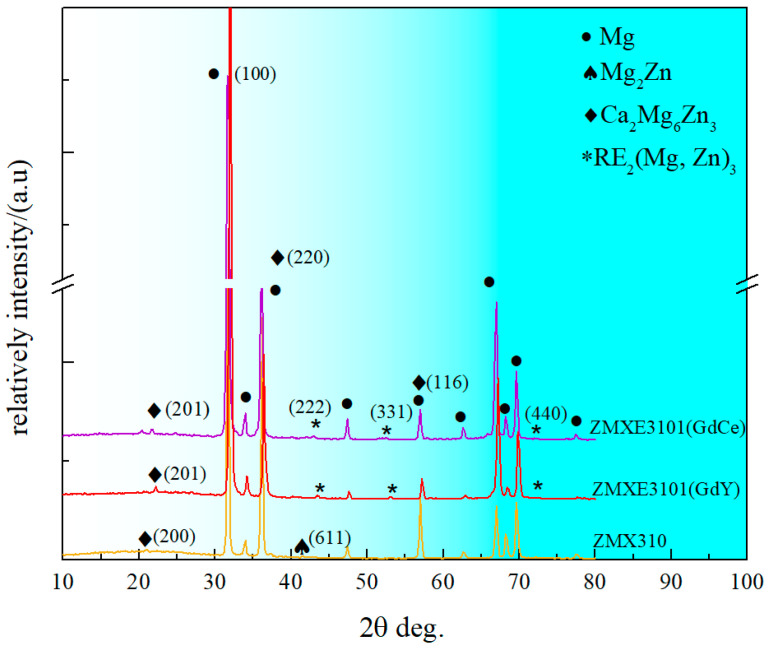
XRD patterns of extruded samples.

After undergoing the extrusion deformation process, the second phases within the as-cast samples undergo significant compression, and during this process, the formation of new phases may be induced. In light of this, accurately identifying the specific types of second phases becomes particularly crucial. By the X-ray diffraction (XRD) analysis findings (as shown in Figure 2), the second-phase composition of the ZMX310 alloy primarily consists of Mg_2_Zn_3_Ca and Mg_2_Zn phases, which is consistent with the findings reported in the previous literature [25,26,27]. Meanwhile, in the ZMXE3101(GdCe) alloy and ZMXE3101(GdY) alloy, the Mg_3_(Gd, Ce)_2_Zn_3_ and Mg_3_(Gd, Y)_2_Zn_3_ phases were formed, respectively. Furthermore, in addition to the aforementioned second phase, nanoscale Mn particles and Ca-rich phases should also exist within the alloy. However, these particles are challenging to detect using X-ray diffraction (XRD) and a Scanning Electron Microscope (SEM). Nevertheless, the existence of these phases has been confirmed by a substantial body of literature [13,26,28].

**Figure 3:**
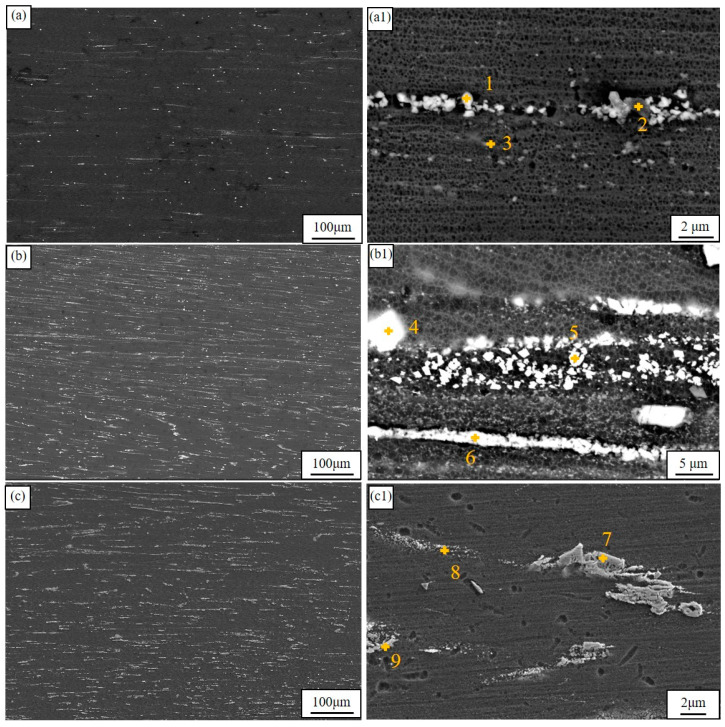
Low- and high-magnification SEM microstructures of as-extruded alloys: (**a**,**a1**) ZMX310; (**b**,**b1**) ZMXE3101(GdCe); (**c**,**c1**) ZMXE3101(GdY).

Figure 3 illustrates the extruded microstructure and typical morphologies of the second phase. The second phases in all alloys are distributed along the direction of extrusion since during the extrusion process, the metal undergoes significant deformation as they are forced by the shear forces through the die opening. In the ZMX310 alloy, numerous finely fragmented second phases are observed, with EDS analysis confirming that the fine fragments are Mg_2_Zn_3_Ca particles and the plate-like phases are Mg_2_Zn particles, as shown in Table 3 for the EDS analysis results. It is worth pointing out that the composition of EDS data does not match the results of XRD very well. This is because EDS requires a very smooth sample surface for accurate quantitative analysis, but the hardness of the second phase is very different from that of the matrix, and the corrosion rate of the matrix and the second phase to the corrosive agent is different. It is difficult to prepare a very smooth sample surface through mechanical polishing and etching. Despite this, EDS can be used for qualitative analysis. In the ZTX310 alloy system, there is a marked augmentation in the abundance of spheroidal and cubic phases, which are unequivocally identified as Mg_2_Zn_3_Ca and Mg_2_Zn, respectively. This reveals that in the ZMXE3101(GdCe) alloy, most of the second phases are fragmented, although a small number of blocky phases are also present, identified as rare earth phases by EDS analysis. In contrast, for the ZMXE3101(GdY) alloy, a portion of the second phase is fragmented, while another portion remains in its original state. The EDS data reveal that the coarse phases are Mg_3_(Gd, Ce)_2_Zn_3_, and the microscale phases are Mg-Zn-Ce-Ca and Mg_2_Zn_3_Ca phases. Overall, the incorporation of RE elements significantly increases the concentration of secondary phases.

**Figure 4:**
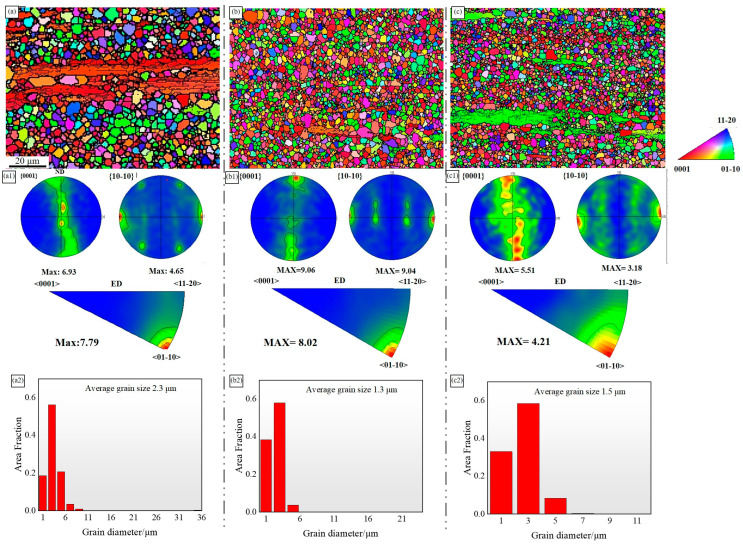
EBSD maps and pole figures of {0001} and {10–10} crystal planes; inverse pole figures of ED and average recrystallized grain sizes: (**a**–**a2**) ZMX310; (**b**–**b2**) ZMXE3101(GdCe); (**c**–**c2**) ZMXE3101(GdY).

Figure 4 displays an Electron Backscatter Diffraction (EBSD) image, accompanied by pole figure information and the distribution of recrystallized grain sizes. The results indicate that none of the alloys underwent complete recrystallization. Among them, the ZMXE3101(GdCe) alloy exhibited the highest degree of recrystallization. The statistically averaged grain sizes were 2.3 μm, 1.3 μm, and 1.5 μm. The combined addition of Gd-Ce and Gd-Y significantly refined the grain structure. SEM images (Figure 4(b2)) reveal that during deformation, the ZMXE3101(GdCe) alloy exhibited the finest grain size, compared to the ZMXE3101(GdY) alloy, suggesting that more fractured hard second phase constituents in ZMXE3101(GdCe) alloy more effectively promoted recrystallization nucleation in the magnesium alloy, enhancing its nucleation rate. Conversely, the coarse-grained secondary phase particles formed in the ZMXE3101(GdY) alloy effectively promoted the level of recrystallization of the alloy on a more macroscopic scale. Crystallographic pole figures and their inverse counterparts derived from the EBSD data indicate that the fibrous structures of all alloys exhibited a <10-10>//ED deformation texture. After doping with RE elements, the matrix texture of the ZMXE3101(GdY) alloy decreased, while that of the ZMXE3101(GdCe) alloy increased. An increase in the matrix texture is beneficial for enhancing strength but may decrease the alloy’s ductility.

The precipitates can potentially influence recrystallization and grain growth through particle-stimulated nucleation (PSN) or by pinning dislocations to hinder the migration of grain boundaries [13]. The EBSD images show that the area of unrecrystallized grains in ZMX310 is significantly higher than that of the other two alloys. This is because the coarse phase particles composed of rare earths often have a particle size greater than 1 μm, which provides PSN sites during deformation and plays an important role in weakening the texture and promoting recrystallization. On the other hand, nanoscale precipitates such as Mn particles and nano-sized Ca-rich phases inhibit this recrystallization process, as disperse Mn nanophases can impede the motion of low-angle grain boundaries, thereby preventing the growth of newly formed DRXed (dynamically recrystallized) grains. A comparative analysis of the two alloys, ZMXE3101(GdCe) and ZMXE3101(GdY), reveals that, based on SEM morphology, the density of rare earth phases in ZMXE3101(GdCe) is notably higher than that in ZMXE3101(GdY). This higher density facilitates dynamic recrystallization in ZMXE3101(GdCe), leading to the highest degree of recrystallization.

### 3.2. Mechanical Properties

**Figure 5:**
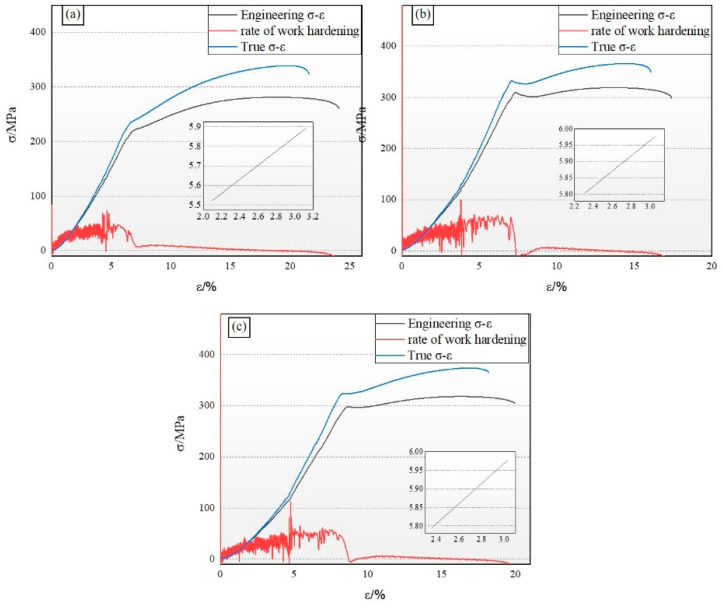
Tensile engineering stress–strain curves. (**a**) ZMX310; (**b**) ZMXE3101(GdCe); (**c**) ZMXE3101(GdY).

It is particularly crucial to gain a deep understanding of the relationship between the true stress–strain curve and the engineering stress–strain curve when analyzing the stress–strain characteristics of magnesium alloys. Figure 5 presents a comparison of the true stress–strain curves and the engineering stress–strain curve for various test specimens at room temperature. It is observable from the figures that, at the commencement of the stress–strain curves, the two curves nearly coincide. However, as strain increases, the actual stress gradually and substantially surpasses the engineering stress. This discrepancy arises because the calculation of true stress incorporates the reduction in the cross-sectional area due to the necking effect of the material, which is not considered in the calculation of engineering stress. Furthermore, the curve of the work-hardening rate offers insightful data concerning the progression rate of material hardening.

The strain-hardening exponent serves as an indicator of a material’s ability to achieve strain homogenization [24]. To further quantify the strain-hardening characteristics of the as-extruded Mg-Sn alloys, the uniform plastic deformation stage in the uniaxial tensile curve is modeled using the Hollomon equation [25]:

$$\sigma =K\varepsilon_{t}^{n}$$

where K is the strength coefficient, $\varepsilon_{t}$ is the true strain in the plastic deformation stage, and n is the strain-hardening exponent. The corresponding data are presented in Table 4.

In this academic study, the ZMX310 alloy is characterized by an engineering design yield stress of 224.6 MPa, a maximum tensile strength of 281.3 MPa, and an elongation rate of 24.5%, demonstrating robust plastic deformation capabilities. The work-hardening exponent (n) is determined to be 0.36, and the material’s strength coefficient (K) is 4.77. This indicates that the specimen maintains favorable secondary processing characteristics and strain capacity throughout the deformation process.

The ZMXE3101(GdCe) alloy exhibits engineering yield and maximum tensile strengths of 304.5 MPa and 319.1 MPa, respectively, accompanied by a decline in elongation to 17.4%. Analysis of the basal pole figure data obtained via Electron Backscatter Diffraction (EBSD) reveals that, despite having an average grain size substantially smaller than the ZMX310 alloy, the ZMXE3101(GdCe) alloy displays a pronounced increase in basal texture. This enhancement fortifies the alloy along the extrusion direction, leading to a reduction in its plastic deformation capability. Upon the addition of Gd-Y, a notable increase in yield strength is observed across the alloys. Specifically, the yield strength of the ZMX310 alloy is elevated by approximately 18%, while the ZTX310 alloy experiences a significant enhancement in yield strength, attaining a value of 296.8 MPa. The ultimate tensile strength of the ZTX310 alloy closely matches that of the ZMXE3101(GdCe) alloy, at 318.3 MPa. Furthermore, the work-hardening exponent (n) of the ZMXE3101(GdY) alloy surpasses that of the ZMXE3101(GdCe) alloy, suggesting that the ZMXE3101(GdY) alloy possesses exceptional comprehensive mechanical properties, such as ductility and secondary deformation capability. Notably, despite the presence of recrystallized grains, our previous research in Mg-Zn alloys [13] indicates that such grains do not invariably result in a decrement in plasticity. The weakened deformation texture within the ZMXE3101(GdY) alloy enhances its plasticity, while the finer grains and elongated unrecrystallized grains collectively contribute to its substantial strength.

### 3.3. Electrochemical Behavior

**Figure 6:**
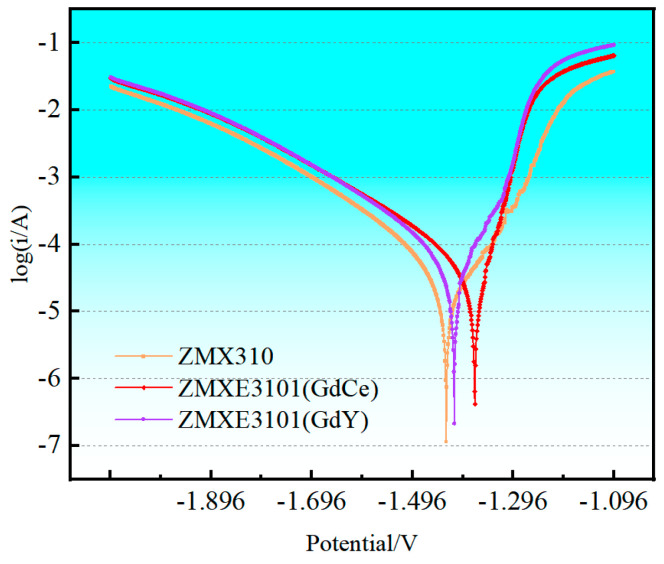
Potentiodynamic Polarization curves.

Figure 6 depicts the polarization characteristic curves of three alloys in a 3.5 wt.% NaCl solution, with the density of corrosion current and corrosion potentials of each alloy presented in Table 5. It is evident that the polarization curves for each of the three alloys display asymmetry, where the anodic segment is markedly steeper compared to the cathodic segment, suggesting a pronounced anodic reaction. As indicated in Table 5, the corrosion current density of the ZMX310 alloy is 14 μA·cm^−2^. The doping of RE elements into the ZMX310 alloy leads to a significant enhancement in corrosion potential, particularly when GdCe elements are incorporated, which leads to a significant elevation in corrosion potential. However, there is an increase in corrosion current density, particularly notable in the case of the ZMXE3101(GdY) alloy.

**Figure 7:**
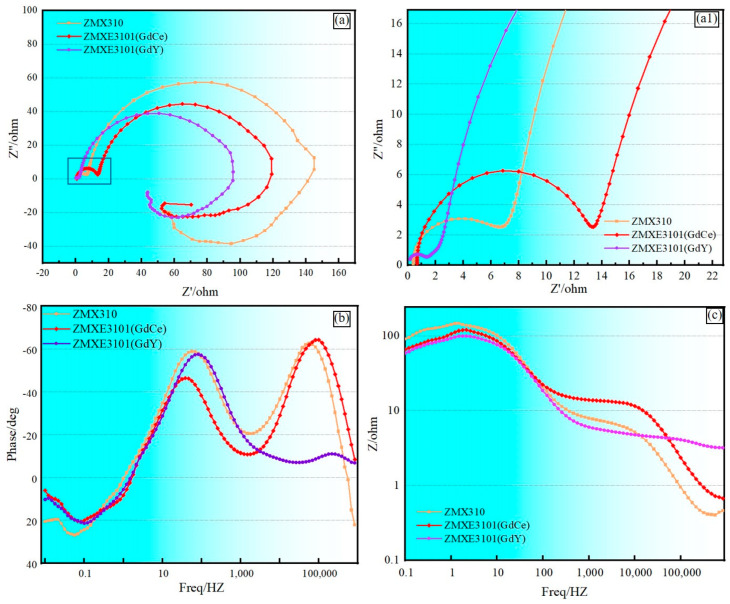
Electrochemical behavior. (**a**,**a1**) EIS Nyquist plots; (**b**) Bode phase plots; (**c**) Bode modulus plots.

In NaCl solution, the corrosion characteristics of extruded magnesium alloys were systematically examined through Electrochemical Impedance Spectroscopy (EIS). Figure 7 illustrates the Nyquist and Bode plots obtained for three distinct alloy compositions. The largest capacitive loop of ZMX310 in Figure 7a indicates that ZMX310 demonstrated superior corrosion resistance, aligning with the findings derived from polarization curve analysis. To elucidate the transient electrochemical behavior at the interface, results of the quantitative analysis of EIS are shown in Table 6.

As shown in Figure 7a, the presence of tree-time constants corresponded to two capacitive loops in the high-frequency capacitive circuit, mid-frequency capacitive circuit, and low-frequency inductive circuit. The two inductive circuit loops represent a capacitive circuit in the high-frequency region corresponding to the electrochemical degradation process of the sample, and another capacitive circuit in the low-frequency region corresponding to the surface film that hinders the corrosion process of the sample [29,30].

Figure 7b,c depict Bode plots illustrating the variation in |Z| (magnitude) and phase angle (θ) with frequency, respectively. Generally, higher phase angle values and |Z| magnitudes indicate better corrosion resistance of the alloy. Among the alloys, at high-frequency ranges, the ZMXE3101(GdCe) alloy exhibits the largest capacitive loop radius, whereas the ZMXE3101(GdY) alloy displays a significantly smaller capacitive loop radius, indicating a superior protective effect of Gd-Ce on magnesium alloys and a far more fragile protective film by Gd-Y compared to ZMX310 at high frequencies. Furthermore, Figure 7c illustrates that the phase angle of the ZMXE3101(GdY) alloy is substantially inferior to that of the other two alloys within the high-frequency domain. Conversely, in the mid-frequency range, the ZMX310 alloy demonstrates the largest capacitive loop radius. Extensive previous research has demonstrated that the incorporation of small amounts of rare earth elements is beneficial for enhancing the corrosion resistance of Mg alloys [31,32,33]. In the present study, it is evident that the added rare earth elements formed a substantial quantity of rare earth phases. These rare earth phases can be regarded as cathodes within the alloy, thereby facilitating the formation of Mg corrosion cells to a certain extent and accelerating the corrosion of Mg alloys.

**Figure 8:**
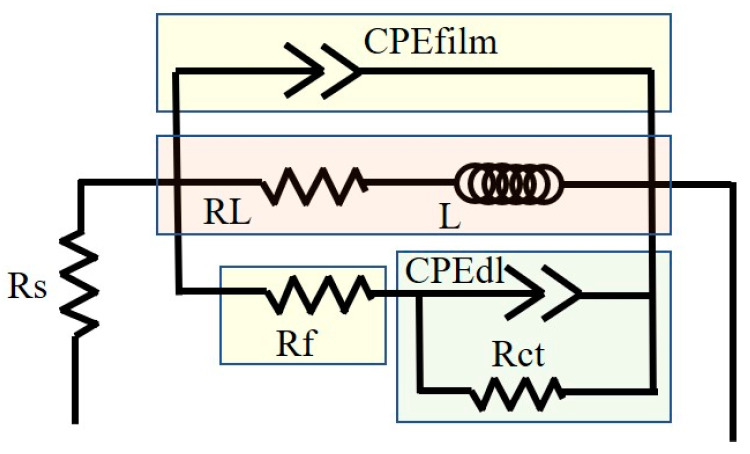
Equivalent circuit model for EIS data fitting.

The similar Nyquist curve shapes for the three alloys indicated that a single current diagram could elucidate the electrochemical impedance characteristics of the double layer and surface film in various alloys. Typically, a larger capacitive loop indicates higher impedance [34]. The equivalent electrical circuit (EEC) for the EIS experiments, shown in Figure 8, includes relevant parameters listed in Table 6, such as ohmic resistance (Rs) due to the electrolyte between the working and reference electrodes. The ideal capacitance element ’C’ is replaced with a constant phase element (CPE), characterized by the parameters Q (non-ideal capacitance) and the exponent n (dispersion index), accounting for the heterogeneity of the electrode surface. The former is influenced by surface defects, impurities, and the presence of second phases. The latter ranges between 0 and 1, with the two extreme values representing purely resistive or purely capacitive behavior, respectively.

The high-frequency capacitance loop results from insufficient protection by the deposited film, as described by CPEdl. Rct represents the resistance caused by charge transfer associated with the interfacial reaction between the substrate and NaCl solution. Rct is crucial for the coupling of hydrogen evolution at the cathode with the ionization of Mg at the anode. Rf represents the film resistance of the corrosion product layer, which can inhibit the diffusion of ions at the interface between the solution and the alloy surface. The CPE film represents the formed film, while the mid-frequency capacitive loop is attributed to the double layer of Mg oxides like Mg(OH)_2_ and MgO at the Mg-NaCl solution interface.

In magnesium alloys, as the frequency decreases, the capacitive loop gradually weakens and transitions into an inductive loop, indicating the systematic removal or breakdown of the corrosion product layer. This may be due to stress during growth and simultaneous H_2_ evolution at the interface [35,36,37,38]. In the low-frequency region, L signifies the inductance, while RL represents the inductive resistance, which indicates the breakdown of a portion of the corrosion product film, leading to the initiation of localized pitting corrosion. An increase in the values of RL and L indicates the formation of a denser corrosion product film on the surface of the alloy addition, effectively inhibiting localized corrosion, From this point, it can be seen that during the corrosion process, the ZMX310 alloy can form a dense protective film, followed by the ZMXE3101(GdCe) alloy, while the protective film of the ZMXE3101(GdY) alloy is the most fragile.

### 3.4. Conclusions

Through comparative studies, the impacts of Gd-Ce and Gd-Y on the mechanical properties, the evolution of the microstructure, and the electrochemical behavior of Mg-Zn-Mn-Ca alloys were analyzed. The outcomes of the research have led to the following conclusions:

(1) The addition of composite rare earths significantly refines the grain size of Mg-Zn-Mn-Ca alloys. Under the extrusion conditions employed in this study, the addition of Gd-Ce effectively promotes alloy recrystallization but increases the intensity of deformation texture components. In contrast, the addition of Gd-Y significantly refines the microstructure of the alloy but attenuates its deformation texture.
(2) For Mg-3Zn-0.5Ca-0.5Mn alloy, the simultaneous addition of Gd-Ce and Gd-Y substantially enhances both the yield and tensile strengths of the alloy, with comparable improvement levels. However, the addition of Gd-Y results in less sacrifice of plasticity in Mg-3Zn-0.5Ca-0.5Mn alloy, which is attributed to its finer recrystallized grain size and weaker deformation texture.
(3) The addition of both Gd-Ce and Gd-Y increases the corrosion current of magnesium alloys. However, Mg-Zn-Mn-Ca alloy with Gd-Y exhibits better corrosion resistance. As revealed by EIS analysis, this is because the addition of Gd-Ce forms a capacitor loop with greater impedance during corrosion, indicating the formation of a protective layer, whereas the addition of Gd-Y hardly forms such a protective layer.

## Figures and Tables

**Table 1:** The chemical compositions of the alloys were determined by ICP-AES (Inductively Coupled Plasma–Atomic Emission Spectrometry).

Alloy	Nominal Composition (wt.%)	Actual Composition (wt.%)						
		Mg	Zn	Ca	Mn	Gd	Ce	Y
ZMX310	Mg-3Zn-1Mn-0.5Ca	Balance	2.95	0.41	0.85	-	-	-
ZMXE3101(Gd Ce)	Mg-3Zn-1Mn-0.5Ca-1Gd-0.5Ce	Balance	3.21	0.55	0.89	1.10	0.53	-
ZTXE3101(Gd Y)	Mg-3Zn-1Sn-0.5Ca-1Gd-0.5Y	Balance	3.20	0.47	1.12	0.96	-	0.61

**Table 2:** EDS results (wt.%) for Figure 1.

Alloy	Mg	Ca	Zn	Mn	Gd	Ce	Y
ZMX310							
1	43.9	15.0	40.9	0.2	-	-	-
2	60.8	9.7	29.4	-	-	-	-
ZMXE3101(GdCe)							
3	10.4	-	34.5	-	45.2	9.6	-
4	25.6	3.3	61.3	-	-	-	-
5	27.9	-	51.4	-	-	14.8	-
6	29.9	3.9	44.6	-	-	16.9	-
ZTXE3101(GdY)							
7	19.4	3.5	66.6	-	-	-	-
8	84.0	-	16.0	-	-	-	-
9	11.0	1.91	39.3	-	35.8	-	12.6
10	19.0	6.1	65.3	-	-	-	-

**Table 3:** EDS results (wt.%) for Figure 3.

Alloy	Mg	Ca	Zn	Mn	Gd	Ce	Y
ZMX310							
1	43.9	15.0	40.9	0.2	-	-	-
2	60.8	9.7	29.4	-	-	-	-
3	55.93	-	25.40	-	-	-	-
ZMXE3101(GdCe)							
4	45.0	1.07	34.5	-	23.5	3.0	-
5	30.2	2.1	51.6	-	-	12.95	-
6	32.6	1.6	43.3	4.8	-	14.9	-
ZTXE3101(GdY)							
7	13.1	-	36.5	-	38.3	-	10.4
8	51.5	3.9	42.6	-	-	-	-
9	2.5	1.9	1.00	-	47.7	-	42.9

**Table 4:** Yield strength, tensile strength, elongation, strength coefficient, and strain-hardening exponent.

Alloys	TYS	UTS	δT	K	n
	(MPa)	(MPa)	(%)		
ZMX310	224.6	281.3	24.5	4.77	0.36
ZMXE3101(GdCe)	304.5	319.1	17.4	5.27	0.23
ZMXE3101(GdY)	296.8	318.3	20.3	5.16	0.27

**Table 5:** The fitting results of polarization curves.

Alloy	E_corr_ (V)	I_corr_ (μA·cm^−2^)
ZMX310	−1.419 ± 0.016	14.005 ± 4.52
ZMXE3101(GdCe)	−1.349 ± 0.015	17.095 ± 0.67
ZMXE3101(GdY)	−1.409 ± 0.025	25.689 ± 0.36

**Table 6:** Fitting results of the EIS spectra.

Alloy	Rs (Ω cm^2^)	Q film (×10^−5^ F s^n1^ cm^−2^)	n1	RL (Ω cm^2^)	Q dl (×10^−5^ F s^n2^ cm^−2^)	n2	Rf (Ω cm^2^)	Rct (Ω cm^2^)	L (H·cm^2^)
ZMX310	0.452	0.2074	1.00	83.79	14.49	0.92	6.74	131.70	306.90
ZMXE3101(GdCe)	0.6775	0.0748	1.00	0.03585	13.14	0.98	5.58	86.90	634.4
ZMXE3101(GdY)	2.805	5.776	0.671	78.14	14.57	0.922	2.47	93.05	132.2

## Data Availability

The original contributions presented in the study are included in the article, further inquiries can be directed to the corresponding author.
